# Supplementary material for: Complexome profiling of the Chlamydomonas psb28 mutant reveals TEF5 as an early PSII assembly factor
Source: Plant Cell. 2025 Mar 17;37(6):koaf055. doi: 10.1093/plcell/koaf055 (PMC12164590; doi:10.1093/plcell/koaf055)
Supplement: koaf055_Supplementary_Data [file koaf055_supplementary_data.zip › psb28_interactive.zip!/plots/intensity/qualityPlots/peptides/FMG2_Cre09.g392867.t1.1.html]

### Cre09.g392867.t1.1

Trivial name: FMG2  
SeqCoverage [median]: 12.30  
Mass: 432.52 kDa  
  
 

| Sequence | MissedCleavage | Mass [Da] | wtIntensity | muIntensity | Score |
| --- | --- | --- | --- | --- | --- |
| AASDTGPAYK | 0 | 979.46 | 7645.58 | 5155.68 | 140.35 |
| AGQSLLK | 0 | 715.42 | 6563.26 | 6853.57 | 170.73 |
| AGQSLLKADSATAANR | 1 | 1572.82 | 827.35 | 0.00 | 78.69 |
| AGSASGTAIGAASTVTIKPTFVSAVATGPR | 1 | 2745.47 | 0.00 | 2336.33 | 31.11 |
| AGSLAFVAK | 0 | 862.49 | 9459.22 | 6697.89 | 130.27 |
| AGTAAPISPAYLTSAAAR | 0 | 1687.89 | 9994.40 | 11977.41 | 138.42 |
| AIASNQVEIK | 0 | 1071.59 | 5561.24 | 8526.51 | 110.76 |
| ALATAAVIVPNLYK | 0 | 1442.85 | 9491.03 | 0.00 | 182.71 |
| ASATPTTTR | 0 | 904.46 | 11961.47 | 4525.71 | 133.86 |
| ATPYAGTDAVNIAAANSLAATTNPLK | 0 | 2515.29 | 4023.77 | 0.00 | 72.94 |
| ATSTSVVVVSLPAASTFVK | 0 | 1863.04 | 2924.79 | 0.00 | 105.14 |
| DTNALLLAGSANTAPAYK | 0 | 1789.92 | 1071.56 | 4535.96 | 106.17 |
| GAATAAAVANTSPK | 0 | 1228.64 | 9494.32 | 8181.92 | 163.78 |
| GAQNALTTTTGSK | 0 | 1248.63 | 11037.48 | 8802.28 | 196.88 |
| GSALTISEAK | 0 | 975.52 | 10880.36 | 9120.79 | 113.71 |
| GTSSTGALYQPVSGATAATIQPTIGAVQVTTSTR | 0 | 3291.69 | 0.00 | 1458.97 | 54.43 |
| GVSVVVVATSSADATK | 0 | 1489.80 | 0.00 | 1299.33 | 140.14 |
| GVVLNTASPIYK | 0 | 1260.71 | 13939.21 | 19070.15 | 172.98 |
| IDVGFGNSANLR | 0 | 1261.64 | 4431.66 | 7399.27 | 157.68 |
| INILASNTDSANTDK | 0 | 1575.77 | 348.00 | 561.71 | 130.56 |
| IVSASATGAR | 0 | 931.51 | 12621.10 | 6266.71 | 128.00 |
| IVTFTPAK | 0 | 875.51 | 5870.01 | 4861.83 | 81.30 |
| RMPVVIEPR | 1 | 1095.62 | 8763.09 | 4553.73 | 59.07 |
| SDQTLLK | 0 | 803.44 | 3387.80 | 0.00 | 132.08 |
| SGSATAGLVK | 0 | 889.49 | 6868.74 | 6772.86 | 143.70 |
| SSALATGPSYVPK | 0 | 1276.67 | 11595.25 | 7003.96 | 194.54 |
| STNTALR | 0 | 761.40 | 4262.28 | 5344.68 | 134.06 |
| TIVVALPIQSK | 0 | 1167.72 | 20365.07 | 27786.09 | 132.88 |
| VGGVGGTNLVPYPTATTISPR | 0 | 2056.10 | 2473.40 | 3267.68 | 53.75 |
| VGGVGGTNLVPYPTATTISPRPFGAK | 1 | 2556.37 | 10305.47 | 22317.73 | 93.41 |
| VGSTLIVKPTIASAK | 1 | 1483.90 | 19221.66 | 16714.97 | 189.86 |
| VGTNVQALYVPR | 0 | 1315.72 | 13609.27 | 17848.74 | 242.49 |
| VIPSGSTIAR | 0 | 999.57 | 8173.06 | 5602.80 | 68.22 |
| VTLPVPITLIK | 0 | 1192.78 | 720.03 | 0.00 | 61.76 |
| YLVATTSSSGQAYIAR | 0 | 1686.86 | 603.15 | 403.91 | 101.39 |
|  |  |  | 248494.1 | 235249.2 |  |
